# From Genes, Proteins, and Clinical Manifestation: Why Do We Need to Better Understand Age-Related Macular Degeneration?

**DOI:** 10.1016/j.xops.2022.100174

**Published:** 2022-05-31

**Authors:** Steffen Schmitz-Valckenberg, Moussa A. Zouache, Gregory S. Hageman, Monika Fleckenstein

The term *age-related macular degeneration* (AMD) was introduced in 1984.^1^ Before this, terms such as *senile (disciform) macular degeneration*, *disciform degeneration*, *involutional macular degeneration*, *senile amblyopia*, and *Kuhnt-Junius macular degeneration* were used.^2^ Clinicians quickly adopted the term *AMD* and have applied it all too frequently, going as far as using it to describe any degeneration of the macula in individuals older than 50 to 60 years, provided that no other distinctive cause is evident. Determining the causes of a broadly defined multifactorial disease without attempting to further classify its clinical manifestations is a far too simplistic approach and has likely hampered progress in AMD research.^3^ It has now been > 15 years since 2 major genetic risk loci were shown to be associated with risk of AMD developing, one in the *CFH-CFHR5* region on chromosome 1q32 and the other in the *ARMS2/HTRA1* locus on chromosome 10q26. Yet, the importance of genetic risk factors in the clinical manifestations and progression of AMD are largely ignored.^4^, ^5^, ^6^, ^7^, ^8^, ^9^ The consequences of this are far reaching, particularly in clinical trials aiming to investigate innovative therapies for the different forms of AMD. This lack of consideration for the genetic structure of AMD is increasingly difficult to explain as more research results point toward a definitive impact of genetic risk profiles on the molecular mechanisms, manifestation, and risk of progression of this blinding disease.^10^, ^11^, ^12^, ^13^

We should aim to search for relevant features that are associated with characteristic genomic and molecular patterns while advancing in AMD research.

In this issue of *Ophthalmology Science*, Kato et al^14^ present their results after assessment of the association of complement activation products in the aqueous humor of 236 eyes with different forms of macular neovascularization and 49 control eyes. One key strength of this study is that genotypes for 3 loci commonly associated with AMD were available for all participants. Variants considered by the authors included the risk-associated *CFH* Y402H on chromosome 1q32, the risk-associated *ARMS2/HTRA1* A69S on chromosome 10q26, and the protection-conferring *CFH* I62V on chromosome 1q32. The authors report that C3a concentrations in aqueous humor are significantly higher in patients with macular neovascularization carrying the *ARMS2/HTRA1* A69S risk allele as compared with those carrying *CFH* I62 and *CFH* 402H risk alleles. No significant association with genotype could be established with the other complement activation products investigated (C4a and C5a). The authors conclude that their results underscore a role of the *ARMS2* A69S risk allele in local activation of the alternative pathway of the complement system. The results support the hypothesis that the *ARMS2/HTRA1* locus and complement activation play a pivotal role in AMD pathogenesis. They also indicate that genetic risk profiles should be considered to determine best delivery routes and regimen for complement-directed therapeutics.

What can we learn from this report as we move forward to refine AMD research? First, the study design and especially the collection of aqueous humor samples can be well incorporated into clinical research studies and clinical trials. Second, the study underscores that genes, proteins, and the clinical manifestations of AMD can be linked to each other. It is imperative to consider these associations systematically. Third, a need exists for investigating larger cohorts and expanding the assessment of both complement components and their association with genetic variants. Scientists and clinicians should be encouraged to collaborate and combine efforts, including to conduct multicenter clinical research. This will be key to better understanding and elucidating the underlying molecular mechanisms that cause the clinical manifestations of the various forms of AMD. This in turn will be key to developing effective and safe treatments for individuals affected by progressive visual impairment caused by this disease.

The study by Kato et al^14^ also points toward different definitions of clinical AMD, the genetic heterogeneity that likely exists among various populations, and understanding how to apply the collective term *AMD* in distinct geographical regions. The authors emphasize that their study was carried out in Japan, implying that the results may not be directly transferable to other populations. Indeed, an increasing body of evidence suggests that both the genetic structure and the clinical manifestation of AMD in East Asia differ as compared with those in populations of European ancestry.^15^

Genome-wide association studies have identified 34 loci independently associated with AMD among patients of European ancestry; however, not all of these associations could be identified among East Asian populations.^16^^,^^17^ However, genome-wide association studies have confirmed that the most common genetic contributors to disease among both populations are variants associated with the *CFH-CFHR5* and *ARMS2/HTRA1* loci. Because *CFH* 402H is rare among East Asians (frequency, <5%), its contribution to disease in this population is uncommon.^18^ The only variant significantly associated with AMD within *CFH-CFHR5* among East Asians confers a lower risk for disease developing. Therefore, the main gene-directed form of AMD among East Asians is almost exclusively driven by variants associated with the *ARMS2/HTRA1* locus.

Furthermore, it is interesting to note that the authors consider pachychoroid neovasculopathy (PNV) as a phenotype of the neovascular AMD disease spectrum, in addition to drusen-associated neovascular AMD, polypoidal vasculopathy, and retinal angiomatous proliferations. The authors provide detailed definitions of characteristic clinical features of PNV, including the presence or history of central serous chorioretinopathy or pachychoroid pigment epitheliopathy-related abnormalities of the retinal pigment epithelium. We welcome these detailed definitions and the approach by the authors to separately assess these 4 different types of macular neovascularization throughout their analysis. In this context, we believe that it is important to note a lack of consensus regarding whether PNV should be included in the AMD spectrum. Although some overlap may be conceivable, most clinicians, particularly in Western countries, would regard PNV as a different disease entity, also appreciating that PNV is common in various populations with different ancestry backgrounds. Nevertheless, all 4 forms of macular neovascularization included in the study generally demonstrate response to anti–VEGF and associated treatment strategies, allowing clinicians to control the invasion and exudation of abnormal blood vessels. At the same time, evidence is compelling that they manifest and behave differently.^19^ This is relevant for clinical management. Moreover, the differences extend far beyond the pure manifestation of exudation and neovascularization. For example, they also encompass age of onset and phenotypic hallmarks in degeneration of retinal tissue such as drusen phenotype and pigmentary abnormalities. This has been clearly demonstrated by the daily use of high-resolution multimodal imaging. We propose that a clear need exists to better acknowledge these obvious observations in AMD research efforts. Rather than further expanding the use of the term *AMD*, we should aim to understand the various manifestations of retinal degeneration. Indeed, we should aim to search for relevant features that are associated with characteristic genomic and molecular patterns while advancing in AMD research.
